# (*Z*)-3-Methyl-1-phenyl-4-[(*p*-tol­yl)(*p*-tolyl­amino)­methyl­idene]-1*H*-pyrazol-5(4*H*)-one

**DOI:** 10.1107/S1600536812040330

**Published:** 2012-09-29

**Authors:** Naresh Sharma, Komal M. Vyas, R. N. Jadeja, Rajni Kant, Vivek K. Gupta

**Affiliations:** a Post-Graduate Department of Physics & Electronics, University of Jammu, Jammu Tawi 180 006, India; b Department of Chemistry, Faculty of Science, The M. S. University of Baroda, Vadodara 390 002, India

## Abstract

In the title mol­ecule, C_25_H_23_N_3_O_2_, the pyrazole ring forms dihedral angles of 28.56 (7), 80.35 (7) and 31.99 (7)° with the phenyl ring, the *p*-tolyl ring and the *p*-tolyl­amino ring, respectively. The N—H group attached to the exocyclic C=C bond is in a *syn* arrangement with respect to the C=O bond of the pyrazolone group and an intra­molecular N—H⋯O hydrogen bond is observed. In the crystal, weak C—H⋯π inter­actions link mol­ecules along [100].

## Related literature

For related structures, see: Vyas *et al.* (2011 ▶); Ma *et al.* (2006 ▶); Sun *et al.* (2007 ▶).
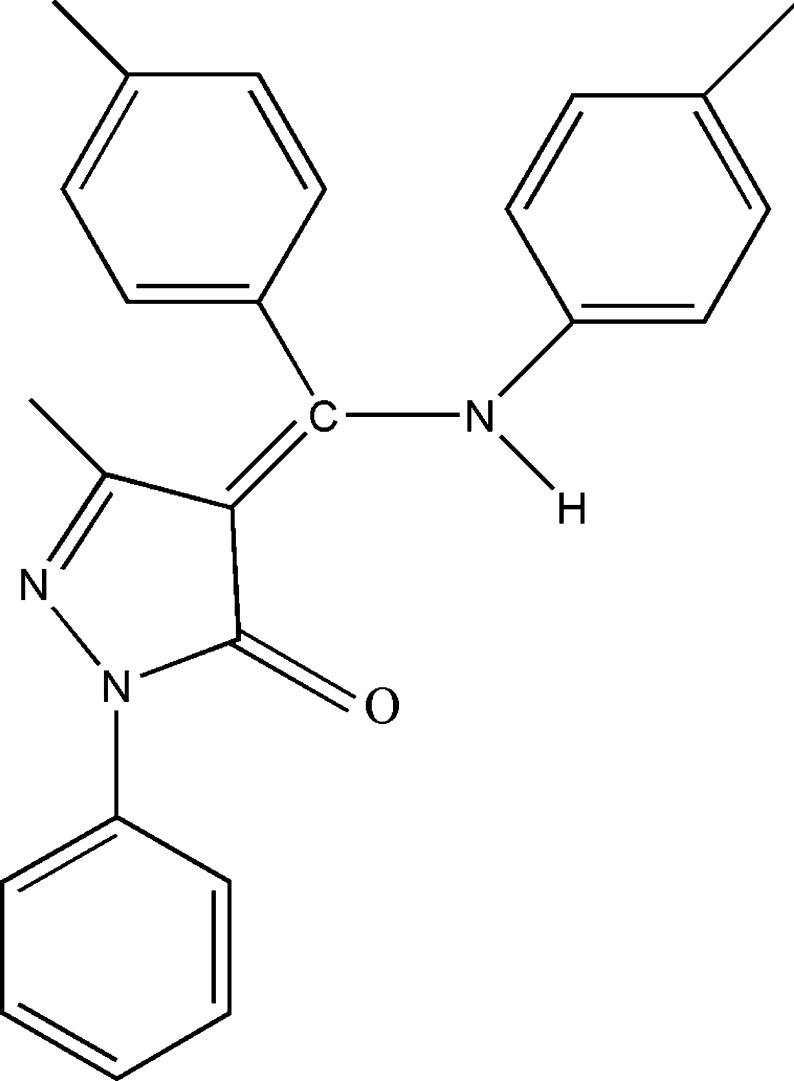


## Experimental

### 

#### Crystal data

C_25_H_23_N_3_O
*M* *_r_* = 381.46
Monoclinic, 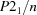

*a* = 9.2694 (4) Å
*b* = 18.3156 (8) Å
*c* = 12.6716 (7) Å
β = 105.124 (5)°
*V* = 2076.80 (17) Å^3^
*Z* = 4
Mo *K*α radiation
μ = 0.08 mm^−1^
*T* = 293 K
0.30 × 0.30 × 0.20 mm

#### Data collection

Oxford Diffraction Xcalibur Sapphire3 diffractometer
Absorption correction: multi-scan (*CrysAlis PRO*; Oxford Diffraction, 2010 ▶) *T*_min_ = 0.914, *T*_max_ = 1.000
9542 measured reflections
4077 independent reflections
2343 reflections with *I* > 2σ(*I*)
*R*_int_ = 0.039

#### Refinement

*R*[*F* ^2^ > 2σ(*F* ^2^)] = 0.052
*wR*(*F* ^2^) = 0.132
*S* = 1.01
4077 reflections
270 parameters
H atoms treated by a mixture of independent and constrained refinement
Δρ_max_ = 0.17 e Å^−3^
Δρ_min_ = −0.14 e Å^−3^

### 

Data collection: *CrysAlis PRO* (Oxford Diffraction, 2010 ▶); cell refinement: *CrysAlis PRO*; data reduction: *CrysAlis PRO*; program(s) used to solve structure: *SHELXS97* (Sheldrick, 2008 ▶); program(s) used to refine structure: *SHELXL97* (Sheldrick, 2008 ▶); molecular graphics: *ORTEP-3* (Farrugia, 1997 ▶); software used to prepare material for publication: *PLATON* (Spek, 2009 ▶).

## Supplementary Material

Crystal structure: contains datablock(s) I, global. DOI: 10.1107/S1600536812040330/lh5535sup1.cif

Structure factors: contains datablock(s) I. DOI: 10.1107/S1600536812040330/lh5535Isup2.hkl

Supplementary material file. DOI: 10.1107/S1600536812040330/lh5535Isup3.cml

Additional supplementary materials:  crystallographic information; 3D view; checkCIF report

## Figures and Tables

**Table 1:** Hydrogen-bond geometry (Å, °) *Cg* is the centroid of the C7–C12 ring

*D*—H⋯*A*	*D*—H	H⋯*A*	*D*⋯*A*	*D*—H⋯*A*
N21—H21⋯O5	0.99 (2)	1.82 (2)	2.702 (2)	146.4 (17)
C15—H15⋯*Cg* ^i^	0.93	2.63	3.470 (2)	152

Symmetry code: (i) 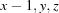.

**Fig. 1.**
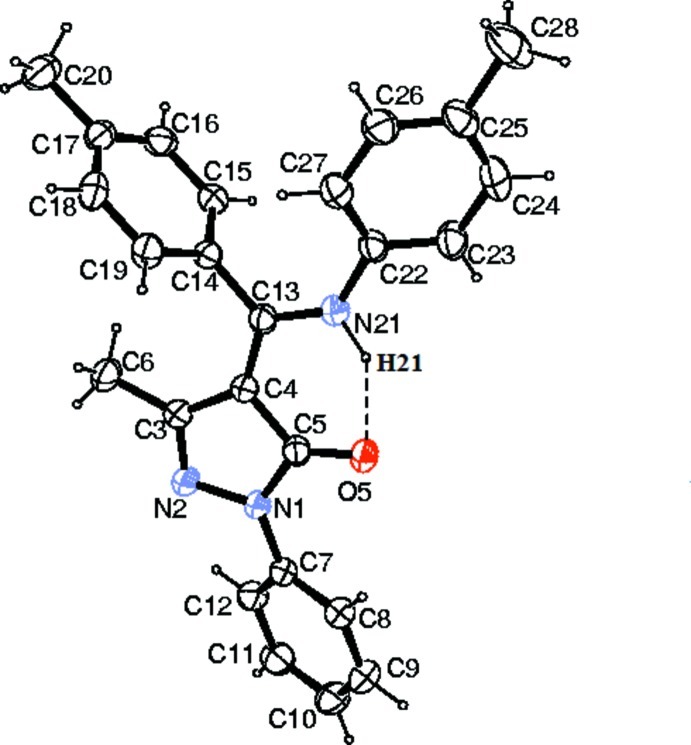
The molecular structure of the title compound with ellipsoids are drawn at the 40% probability level. H atoms are shown as small spheres of arbitrary radii. The dashed line indicates a hydrogen bond.

**Fig. 2.**
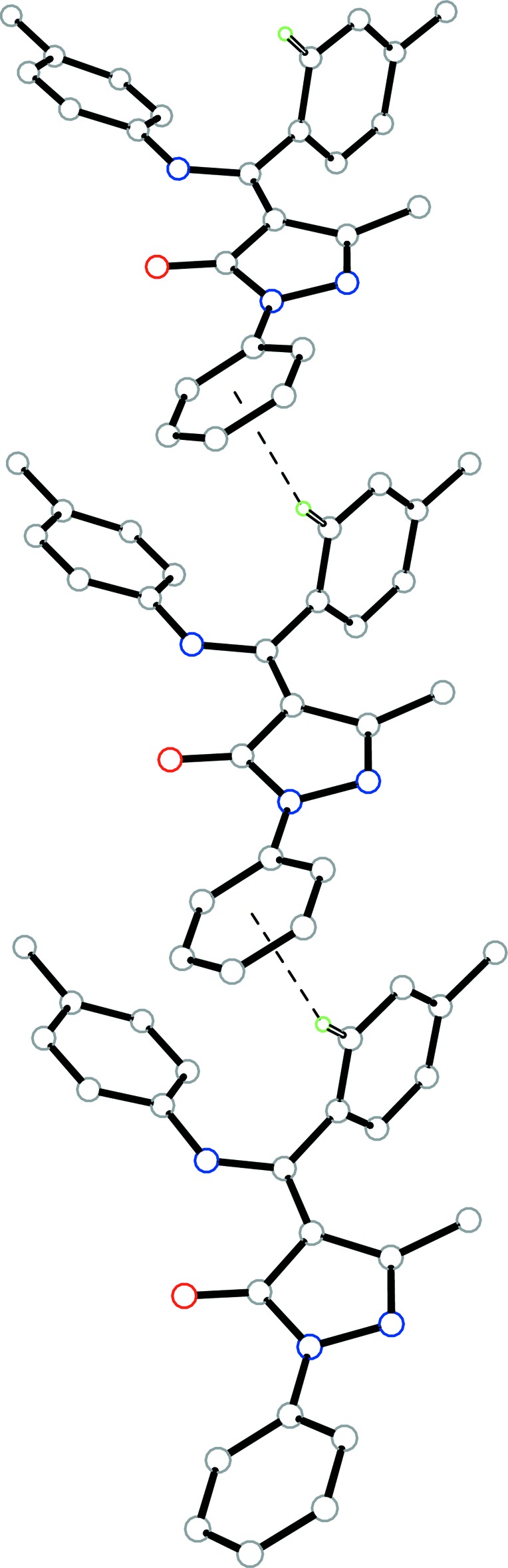
Part of the one-dimensional motif along [100] formed by weak C—H···π interactions (shown as dashed lines).

## supplementary crystallographic information

### Comment

The title compound (I) was prepared as a continuation of our studies of the structures 4-toluoyl pyrazolones (Vyas *et al.*, 2011). The molecular structure of (I) is shown in Fig 1. The bond lengths and angles in (I) are normal and correspond to those observed in related structures (Ma *et al.*, 2006; Sun *et al.*, 2007). The pyrazolone ring forms dihedral angles of 28.56 (7)°, 80.35 (7)° and 31.99 (7)° with the phenyl ring (C7—C12) and two benzene rings (C14—C19 and C22—C27). In The N—H group attached to the exocyclic C═C bond (C4-C13) is in a syn arrangement with respect to the C═O bond of the pyrazolone group and an intramolecular N—H···O hydrogen bond is observed. In the crystal, a weak C—H···π interaction link molecules along [100] (Fig .2).

### Experimental

Equimolar (10 mmol) ethanolic solution (50 ml) of 5-Methyl-4-(4-methyl-benzoyl)-2-phenyl-2,4-dihydro-pyrazol-3-one and *p*-toluidine was refluxed for 6 h in round bottom flask, whereupon a microcrystalline yellow precipitate appeared. The product was then isolated and recrystallized from ethanol, and then dried *in vacuo* to give the title compound in 85% yield. Yellow single crystals suitable for X-ray analysis were obtained by slow evaporation of an ethanol solution of the title compound.

### Refinement

The H atom bonded to N was located in a difference Fourier map and refined isotropically. The remaining H atoms were positioned geometrically and were treated as riding on their parent C atoms, with C—H distances of 0.93–0.96 Å and with *U*_iso_(H) = 1.2*U*_eq_(C) or *U*_iso_(H) = 1.5*U*_eq_(C).

### Crystal data

**Table:**

C_25_H_23_N_3_O	*F*(000) = 808
*M**_r_* = 381.46	*D*_x_ = 1.220 Mg m^−^^3^
Monoclinic, *P*2_1_/*n*	Mo *K*α radiation, λ = 0.71073 Å
Hall symbol: -P 2yn	Cell parameters from 3374 reflections
*a* = 9.2694 (4) Å	θ = 3.5–29.0°
*b* = 18.3156 (8) Å	µ = 0.08 mm^−^^1^
*c* = 12.6716 (7) Å	*T* = 293 K
β = 105.124 (5)°	Block, yellow
*V* = 2076.80 (17) Å^3^	0.30 × 0.30 × 0.20 mm
*Z* = 4	

### Data collection

**Table:**

Oxford Diffraction Xcalibur Sapphire3 diffractometer	4077 independent reflections
Radiation source: fine-focus sealed tube	2343 reflections with *I* > 2σ(*I*)
Graphite monochromator	*R*_int_ = 0.039
Detector resolution: 16.1049 pixels mm^-1^	θ_max_ = 26.0°, θ_min_ = 3.5°
ω scans	*h* = −11→11
Absorption correction: multi-scan (*CrysAlis PRO*; Oxford Diffraction, 2010)	*k* = −22→19
*T*_min_ = 0.914, *T*_max_ = 1.000	*l* = −15→12
9542 measured reflections	

### Refinement

**Table:**

Refinement on *F*^2^	Secondary atom site location: difference Fourier map
Least-squares matrix: full	Hydrogen site location: inferred from neighbouring sites
*R*[*F*^2^ > 2σ(*F*^2^)] = 0.052	H atoms treated by a mixture of independent and constrained refinement
*wR*(*F*^2^) = 0.132	*w* = 1/[σ^2^(*F*_o_^2^) + (0.0435*P*)^2^] where *P* = (*F*_o_^2^ + 2*F*_c_^2^)/3
*S* = 1.01	(Δ/σ)_max_ = 0.001
4077 reflections	Δρ_max_ = 0.17 e Å^−^^3^
270 parameters	Δρ_min_ = −0.14 e Å^−^^3^
0 restraints	Extinction correction: *SHELXL97* (Sheldrick, 2008), Fc^*^=kFc[1+0.001xFc^2^λ^3^/sin(2θ)]^-1/4^
Primary atom site location: structure-invariant direct methods	Extinction coefficient: 0.0018 (5)

### Special details

**Table:**

Experimental. *CrysAlis PRO*, Oxford Diffraction Ltd., Version 1.171.34.40 (release 27–08-2010 CrysAlis171. NET) (compiled Aug 27 2010,11:50:40) Empirical absorption correction using spherical harmonics, implemented in SCALE3 ABSPACK scaling algorithm.
Geometry. All e.s.d.'s (except the e.s.d. in the dihedral angle between two l.s. planes) are estimated using the full covariance matrix. The cell e.s.d.'s are taken into account individually in the estimation of e.s.d.'s in distances, angles and torsion angles; correlations between e.s.d.'s in cell parameters are only used when they are defined by crystal symmetry. An approximate (isotropic) treatment of cell e.s.d.'s is used for estimating e.s.d.'s involving l.s. planes.
Refinement. Refinement of *F*^2^ against ALL reflections. The weighted *R*-factor *wR* and goodness of fit *S* are based on *F*^2^, conventional *R*-factors *R* are based on *F*, with *F* set to zero for negative *F*^2^. The threshold expression of *F*^2^ > σ(*F*^2^) is used only for calculating *R*-factors(gt) *etc*. and is not relevant to the choice of reflections for refinement. *R*-factors based on *F*^2^ are statistically about twice as large as those based on *F*, and *R*- factors based on ALL data will be even larger.

### Fractional atomic coordinates and isotropic or equivalent isotropic displacement parameters (Å^2^)

**Table:**

	*x*	*y*	*z*	*U*_iso_*/*U*_eq_	
N1	0.37979 (16)	0.27791 (8)	0.08898 (14)	0.0507 (5)	
N2	0.34012 (18)	0.35209 (9)	0.07998 (16)	0.0566 (5)	
C3	0.1970 (2)	0.35428 (11)	0.03270 (17)	0.0497 (6)	
C4	0.1351 (2)	0.28261 (10)	0.01024 (16)	0.0453 (5)	
C5	0.2589 (2)	0.23347 (11)	0.04912 (16)	0.0466 (5)	
O5	0.26105 (14)	0.16518 (7)	0.04952 (12)	0.0560 (4)	
C6	0.1232 (2)	0.42734 (11)	0.0119 (2)	0.0711 (8)	
H6A	0.1927	0.4645	0.0460	0.107*	
H6B	0.0380	0.4284	0.0416	0.107*	
H6C	0.0914	0.4361	−0.0654	0.107*	
C7	0.5268 (2)	0.25907 (11)	0.14877 (18)	0.0489 (5)	
C8	0.5900 (2)	0.19386 (12)	0.12908 (19)	0.0588 (6)	
H8	0.5369	0.1621	0.0757	0.071*	
C9	0.7325 (2)	0.17612 (13)	0.1892 (2)	0.0707 (7)	
H9	0.7753	0.1322	0.1761	0.085*	
C10	0.8117 (3)	0.22272 (15)	0.2681 (2)	0.0741 (8)	
H10	0.9072	0.2102	0.3089	0.089*	
C11	0.7492 (2)	0.28799 (14)	0.28674 (19)	0.0656 (7)	
H11	0.8033	0.3198	0.3397	0.079*	
C12	0.6064 (2)	0.30674 (12)	0.22733 (18)	0.0562 (6)	
H12	0.5644	0.3509	0.2401	0.067*	
C13	−0.0104 (2)	0.25866 (11)	−0.03560 (16)	0.0447 (5)	
C14	−0.1359 (2)	0.31073 (10)	−0.07300 (16)	0.0426 (5)	
C15	−0.2309 (2)	0.32528 (11)	−0.00777 (18)	0.0503 (6)	
H15	−0.2187	0.3008	0.0583	0.060*	
C16	−0.3446 (2)	0.37642 (12)	−0.04055 (19)	0.0574 (6)	
H16	−0.4067	0.3861	0.0047	0.069*	
C17	−0.3674 (2)	0.41302 (11)	−0.1384 (2)	0.0541 (6)	
C18	−0.2724 (2)	0.39762 (12)	−0.20344 (18)	0.0593 (6)	
H18	−0.2858	0.4217	−0.2699	0.071*	
C19	−0.1579 (2)	0.34719 (11)	−0.17182 (17)	0.0555 (6)	
H19	−0.0956	0.3377	−0.2170	0.067*	
C20	−0.4894 (2)	0.46931 (12)	−0.1720 (2)	0.0826 (9)	
H20A	−0.5745	0.4544	−0.1474	0.124*	
H20B	−0.5176	0.4739	−0.2502	0.124*	
H20C	−0.4538	0.5155	−0.1398	0.124*	
N21	−0.0338 (2)	0.18632 (9)	−0.04121 (15)	0.0546 (5)	
C22	−0.1653 (2)	0.14481 (11)	−0.08416 (18)	0.0503 (6)	
C23	−0.1729 (3)	0.07733 (11)	−0.03820 (19)	0.0636 (7)	
H23	−0.0940	0.0612	0.0186	0.076*	
C24	−0.2965 (3)	0.03342 (13)	−0.0756 (2)	0.0750 (8)	
H24	−0.2995	−0.0122	−0.0440	0.090*	
C25	−0.4156 (3)	0.05601 (13)	−0.1591 (2)	0.0666 (7)	
C26	−0.4042 (3)	0.12227 (13)	−0.2062 (2)	0.0676 (7)	
H26	−0.4824	0.1379	−0.2639	0.081*	
C27	−0.2809 (2)	0.16660 (12)	−0.17105 (19)	0.0621 (7)	
H27	−0.2756	0.2109	−0.2057	0.074*	
C28	−0.5540 (3)	0.00866 (15)	−0.1984 (3)	0.1072 (11)	
H28A	−0.5999	0.0188	−0.2741	0.161*	
H28B	−0.6235	0.0192	−0.1560	0.161*	
H28C	−0.5261	−0.0419	−0.1900	0.161*	
H21	0.060 (2)	0.1594 (11)	−0.0076 (17)	0.071 (7)*	

### Atomic displacement parameters (Å^2^)

**Table:**

	*U*^11^	*U*^22^	*U*^33^	*U*^12^	*U*^13^	*U*^23^
N1	0.0428 (9)	0.0367 (10)	0.0719 (12)	0.0019 (8)	0.0134 (8)	0.0041 (9)
N2	0.0461 (10)	0.0386 (10)	0.0830 (13)	0.0030 (8)	0.0129 (9)	0.0032 (9)
C3	0.0441 (11)	0.0432 (13)	0.0617 (14)	0.0017 (10)	0.0135 (10)	0.0022 (11)
C4	0.0409 (10)	0.0384 (12)	0.0571 (13)	0.0009 (9)	0.0136 (9)	0.0017 (10)
C5	0.0500 (12)	0.0393 (13)	0.0524 (13)	−0.0001 (10)	0.0167 (10)	0.0026 (11)
O5	0.0573 (9)	0.0382 (9)	0.0702 (10)	0.0036 (7)	0.0126 (8)	0.0043 (8)
C6	0.0557 (13)	0.0414 (13)	0.107 (2)	0.0041 (11)	0.0059 (13)	0.0020 (13)
C7	0.0434 (11)	0.0462 (13)	0.0589 (14)	0.0036 (10)	0.0165 (10)	0.0100 (11)
C8	0.0518 (13)	0.0508 (14)	0.0747 (16)	0.0046 (11)	0.0183 (11)	0.0078 (12)
C9	0.0602 (15)	0.0585 (16)	0.093 (2)	0.0177 (12)	0.0189 (14)	0.0180 (15)
C10	0.0546 (14)	0.0829 (19)	0.0786 (19)	0.0143 (14)	0.0063 (13)	0.0259 (16)
C11	0.0580 (14)	0.0756 (18)	0.0593 (15)	−0.0034 (13)	0.0084 (12)	0.0096 (13)
C12	0.0522 (12)	0.0586 (15)	0.0606 (14)	0.0035 (11)	0.0197 (11)	0.0062 (12)
C13	0.0487 (11)	0.0385 (12)	0.0502 (12)	0.0021 (10)	0.0187 (9)	0.0016 (10)
C14	0.0398 (10)	0.0402 (12)	0.0481 (12)	−0.0007 (9)	0.0119 (9)	−0.0011 (10)
C15	0.0489 (12)	0.0503 (14)	0.0540 (13)	−0.0028 (10)	0.0174 (10)	0.0030 (11)
C16	0.0471 (12)	0.0565 (14)	0.0725 (16)	−0.0016 (11)	0.0227 (11)	−0.0076 (13)
C17	0.0439 (12)	0.0397 (13)	0.0700 (16)	−0.0004 (10)	−0.0006 (11)	−0.0071 (12)
C18	0.0719 (15)	0.0464 (14)	0.0547 (14)	0.0044 (12)	0.0077 (12)	0.0075 (12)
C19	0.0662 (14)	0.0516 (14)	0.0529 (14)	0.0054 (11)	0.0228 (11)	0.0029 (11)
C20	0.0592 (15)	0.0561 (15)	0.116 (2)	0.0091 (12)	−0.0072 (15)	−0.0108 (15)
N21	0.0467 (10)	0.0416 (11)	0.0733 (13)	−0.0009 (9)	0.0117 (9)	0.0031 (10)
C22	0.0532 (12)	0.0380 (12)	0.0631 (14)	−0.0033 (10)	0.0211 (11)	−0.0037 (11)
C23	0.0741 (15)	0.0430 (14)	0.0709 (16)	−0.0051 (12)	0.0139 (13)	−0.0025 (12)
C24	0.1014 (19)	0.0424 (14)	0.0863 (18)	−0.0170 (14)	0.0338 (16)	−0.0045 (14)
C25	0.0684 (15)	0.0524 (15)	0.0857 (18)	−0.0165 (13)	0.0324 (14)	−0.0247 (14)
C26	0.0591 (14)	0.0579 (16)	0.0823 (17)	−0.0048 (12)	0.0120 (12)	−0.0122 (14)
C27	0.0601 (14)	0.0498 (14)	0.0724 (16)	−0.0072 (11)	0.0102 (12)	−0.0001 (12)
C28	0.099 (2)	0.089 (2)	0.140 (3)	−0.0458 (17)	0.0429 (19)	−0.038 (2)

### Geometric parameters (Å, º)

**Table:**

N1—C5	1.370 (2)	C15—H15	0.9300
N1—N2	1.404 (2)	C16—C17	1.377 (3)
N1—C7	1.418 (2)	C16—H16	0.9300
N2—C3	1.306 (2)	C17—C18	1.383 (3)
C3—C4	1.431 (3)	C17—C20	1.507 (3)
C3—C6	1.495 (3)	C18—C19	1.385 (3)
C4—C13	1.392 (2)	C18—H18	0.9300
C4—C5	1.440 (2)	C19—H19	0.9300
C5—O5	1.251 (2)	C20—H20A	0.9600
C6—H6A	0.9600	C20—H20B	0.9600
C6—H6B	0.9600	C20—H20C	0.9600
C6—H6C	0.9600	N21—C22	1.419 (2)
C7—C8	1.381 (3)	N21—H21	0.99 (2)
C7—C12	1.384 (3)	C22—C23	1.376 (3)
C8—C9	1.381 (3)	C22—C27	1.380 (3)
C8—H8	0.9300	C23—C24	1.378 (3)
C9—C10	1.372 (3)	C23—H23	0.9300
C9—H9	0.9300	C24—C25	1.378 (3)
C10—C11	1.375 (3)	C24—H24	0.9300
C10—H10	0.9300	C25—C26	1.369 (3)
C11—C12	1.384 (3)	C25—C28	1.521 (3)
C11—H11	0.9300	C26—C27	1.378 (3)
C12—H12	0.9300	C26—H26	0.9300
C13—N21	1.342 (2)	C27—H27	0.9300
C13—C14	1.483 (2)	C28—H28A	0.9600
C14—C15	1.382 (3)	C28—H28B	0.9600
C14—C19	1.386 (3)	C28—H28C	0.9600
C15—C16	1.390 (3)		
			
C5—N1—N2	111.90 (14)	C17—C16—C15	121.6 (2)
C5—N1—C7	129.18 (16)	C17—C16—H16	119.2
N2—N1—C7	118.26 (15)	C15—C16—H16	119.2
C3—N2—N1	106.27 (15)	C16—C17—C18	117.72 (19)
N2—C3—C4	111.68 (17)	C16—C17—C20	121.0 (2)
N2—C3—C6	118.15 (18)	C18—C17—C20	121.2 (2)
C4—C3—C6	130.17 (17)	C17—C18—C19	121.5 (2)
C13—C4—C3	131.76 (18)	C17—C18—H18	119.2
C13—C4—C5	122.98 (18)	C19—C18—H18	119.2
C3—C4—C5	105.24 (15)	C18—C19—C14	120.2 (2)
O5—C5—N1	125.57 (16)	C18—C19—H19	119.9
O5—C5—C4	129.53 (17)	C14—C19—H19	119.9
N1—C5—C4	104.89 (16)	C17—C20—H20A	109.5
C3—C6—H6A	109.5	C17—C20—H20B	109.5
C3—C6—H6B	109.5	H20A—C20—H20B	109.5
H6A—C6—H6B	109.5	C17—C20—H20C	109.5
C3—C6—H6C	109.5	H20A—C20—H20C	109.5
H6A—C6—H6C	109.5	H20B—C20—H20C	109.5
H6B—C6—H6C	109.5	C13—N21—C22	131.33 (18)
C8—C7—C12	120.25 (18)	C13—N21—H21	110.9 (11)
C8—C7—N1	120.50 (19)	C22—N21—H21	117.8 (11)
C12—C7—N1	119.25 (18)	C23—C22—C27	118.87 (19)
C9—C8—C7	119.6 (2)	C23—C22—N21	116.91 (18)
C9—C8—H8	120.2	C27—C22—N21	124.16 (19)
C7—C8—H8	120.2	C22—C23—C24	120.5 (2)
C10—C9—C8	120.5 (2)	C22—C23—H23	119.7
C10—C9—H9	119.7	C24—C23—H23	119.7
C8—C9—H9	119.7	C25—C24—C23	121.1 (2)
C9—C10—C11	119.8 (2)	C25—C24—H24	119.5
C9—C10—H10	120.1	C23—C24—H24	119.5
C11—C10—H10	120.1	C26—C25—C24	117.6 (2)
C10—C11—C12	120.5 (2)	C26—C25—C28	121.1 (2)
C10—C11—H11	119.7	C24—C25—C28	121.2 (2)
C12—C11—H11	119.7	C25—C26—C27	122.2 (2)
C7—C12—C11	119.3 (2)	C25—C26—H26	118.9
C7—C12—H12	120.3	C27—C26—H26	118.9
C11—C12—H12	120.3	C26—C27—C22	119.6 (2)
N21—C13—C4	117.32 (17)	C26—C27—H27	120.2
N21—C13—C14	121.02 (17)	C22—C27—H27	120.2
C4—C13—C14	121.62 (17)	C25—C28—H28A	109.5
C15—C14—C19	118.75 (19)	C25—C28—H28B	109.5
C15—C14—C13	120.14 (18)	H28A—C28—H28B	109.5
C19—C14—C13	121.09 (19)	C25—C28—H28C	109.5
C14—C15—C16	120.2 (2)	H28A—C28—H28C	109.5
C14—C15—H15	119.9	H28B—C28—H28C	109.5
C16—C15—H15	119.9		
			
C5—N1—N2—C3	−1.5 (2)	C3—C4—C13—C14	0.8 (3)
C7—N1—N2—C3	−173.05 (18)	C5—C4—C13—C14	178.85 (19)
N1—N2—C3—C4	1.1 (2)	N21—C13—C14—C15	77.8 (3)
N1—N2—C3—C6	−179.7 (2)	C4—C13—C14—C15	−99.8 (2)
N2—C3—C4—C13	178.0 (2)	N21—C13—C14—C19	−104.0 (2)
C6—C3—C4—C13	−1.1 (4)	C4—C13—C14—C19	78.3 (3)
N2—C3—C4—C5	−0.4 (2)	C19—C14—C15—C16	−1.0 (3)
C6—C3—C4—C5	−179.4 (2)	C13—C14—C15—C16	177.19 (17)
N2—N1—C5—O5	−178.0 (2)	C14—C15—C16—C17	0.9 (3)
C7—N1—C5—O5	−7.6 (4)	C15—C16—C17—C18	−0.4 (3)
N2—N1—C5—C4	1.2 (2)	C15—C16—C17—C20	−178.89 (18)
C7—N1—C5—C4	171.6 (2)	C16—C17—C18—C19	0.0 (3)
C13—C4—C5—O5	0.1 (4)	C20—C17—C18—C19	178.46 (18)
C3—C4—C5—O5	178.6 (2)	C17—C18—C19—C14	−0.1 (3)
C13—C4—C5—N1	−179.07 (19)	C15—C14—C19—C18	0.6 (3)
C3—C4—C5—N1	−0.5 (2)	C13—C14—C19—C18	−177.60 (18)
C5—N1—C7—C8	33.6 (3)	C4—C13—N21—C22	−178.6 (2)
N2—N1—C7—C8	−156.5 (2)	C14—C13—N21—C22	3.6 (4)
C5—N1—C7—C12	−146.3 (2)	C13—N21—C22—C23	−152.0 (2)
N2—N1—C7—C12	23.6 (3)	C13—N21—C22—C27	30.9 (4)
C12—C7—C8—C9	0.8 (3)	C27—C22—C23—C24	−2.4 (4)
N1—C7—C8—C9	−179.15 (19)	N21—C22—C23—C24	−179.6 (2)
C7—C8—C9—C10	0.0 (4)	C22—C23—C24—C25	−0.5 (4)
C8—C9—C10—C11	−0.7 (4)	C23—C24—C25—C26	2.5 (4)
C9—C10—C11—C12	0.7 (4)	C23—C24—C25—C28	−178.2 (2)
C8—C7—C12—C11	−0.7 (3)	C24—C25—C26—C27	−1.6 (4)
N1—C7—C12—C11	179.17 (19)	C28—C25—C26—C27	179.1 (2)
C10—C11—C12—C7	0.0 (3)	C25—C26—C27—C22	−1.3 (4)
C3—C4—C13—N21	−176.9 (2)	C23—C22—C27—C26	3.3 (4)
C5—C4—C13—N21	1.2 (3)	N21—C22—C27—C26	−179.7 (2)

### Hydrogen-bond geometry (Å, º)

Cg is the centroid of the C7–C12 ring

**Table:**

*D*—H···*A*	*D*—H	H···*A*	*D*···*A*	*D*—H···*A*
N21—H21···O5	0.99 (2)	1.82 (2)	2.702 (2)	146.4 (17)
C15—H15···*Cg*^i^	0.93	2.63	3.470 (2)	152

Symmetry code: (i) *x*−1, *y*, *z*.
